# Holistic process development to mitigate proteolysis of a subunit rotavirus vaccine candidate produced in *Pichia pastoris* by means of an acid pH pulse during fed‐batch fermentation

**DOI:** 10.1002/btpr.2966

**Published:** 2020-02-03

**Authors:** M. Lourdes Velez‐Suberbie, Stephen A. Morris, Kawaljit Kaur, John M. Hickey, Sangeeta B. Joshi, David B. Volkin, Daniel G. Bracewell, Tarit K. Mukhopadhyay

**Affiliations:** ^1^ Department of Biochemical Engineering University College London London United Kingdom; ^2^ Department of Pharmaceutical Chemistry, Vaccine Analytics and Formulation Center University of Kansas Lawrence Kansas

**Keywords:** fed‐batch fermentation, ion exchange chromatography, *Pichia pastoris*, proteolysis, vaccine development

## Abstract

To meet the challenges of global health, vaccine design and development must be reconsidered to achieve cost of goods as low as 15¢ per dose. A new recombinant protein‐based rotavirus vaccine candidate derived from non‐replicative viral subunits fused to a P2 tetanus toxoid CD4(+) T cell epitope is currently under clinical development. We have sought to simplify the existing manufacturing process to meet these aims. To this end, we have taken a holistic process development approach to reduce process complexity and costs while producing a product with the required characteristics. We have changed expression system from *Escherichia coli* to *Pichia pastoris*, to produce a secreted product, thereby reducing the number of purification steps. However, the presence of proteases poses challenges to product quality. To understand the effect of fermentation parameters on product quality small‐scale fermentations were carried out. Media pH and fermentation duration had the greatest impact on the proportion of full‐length product. A novel acidic pH pulse strategy was used to minimize proteolysis, and this combined with an early harvest time significantly increased the proportion of full‐length material (60–75%). An improved downstream process using a combination of CIEX and AIEX to further reduce proteases, resulted in maintaining product quality (95% yield).

## 1 INTRODUCTION

The Ultra Low‐cost Transferable Automated (ULTRA) Platform for Vaccine Manufacturing seeks to combine integrated, automated, modular manufacturing^1^ to achieve the aims of the Bill & Melinda Gates Foundation Grand Challenge—“Innovations in Vaccine Manufacturing for Global Markets”. The stated aim of which is “to create a new manufacturing platform to enable ultra‐low cost, high quality, recombinant subunit vaccines at 15¢ (USD) a dose for global health initiatives.” Our initial model system to demonstrate this approach is the production of a recombinant subunit rotavirus vaccine candidate.

Rotaviruses are one of the most common causes of severe diarrheal disease, which prior to the introduction of an effective vaccination program was estimated to result in 500,000 deaths in infants and children worldwide annually.^2^ This was found to be reduced to around 200,000 deaths annually in 2013 following introduction of vaccination programs, with the majority of these occurring in low‐income countries.^3^ Currently, there are two oral live virus rotavirus vaccines available, Rotarix® (GlaxoSmithKline) and RotaTeq® (Merck) both of which are recommended for inclusion in routine childhood vaccination programs by the World Health Organisation.^4^ Recently, two further vaccines, Rotasil® (Serum Institute of India) and ROTAVAC® (Bharat Biotech), have also been pre‐qualified for use by the WHO. However, the efficacy of these vaccines has been found to be low in some low‐income countries with the highest need.^5^ Other concerns with the use of these oral live virus vaccines include an association with a risk of intussusception^6^ and a potential for re‐assortment of live vaccine stains with circulating wild‐type virus stains.^7^ In addition, the cost of these vaccines is relatively high and is further increased by the requirement for an extensive cold chain during storage and distribution.^8^ To alleviate these issues, new Non Replicative Rotavirus Vaccine (NRRV) based on the VP8 viral proteins from the P[4], P[6], and P[8] serotypes, fused to the P2 universal tetanus toxoid CD4(+) T cell epitope, have been developed and are currently undergoing clinical trials.^9^, ^10^

**Figure 1:**
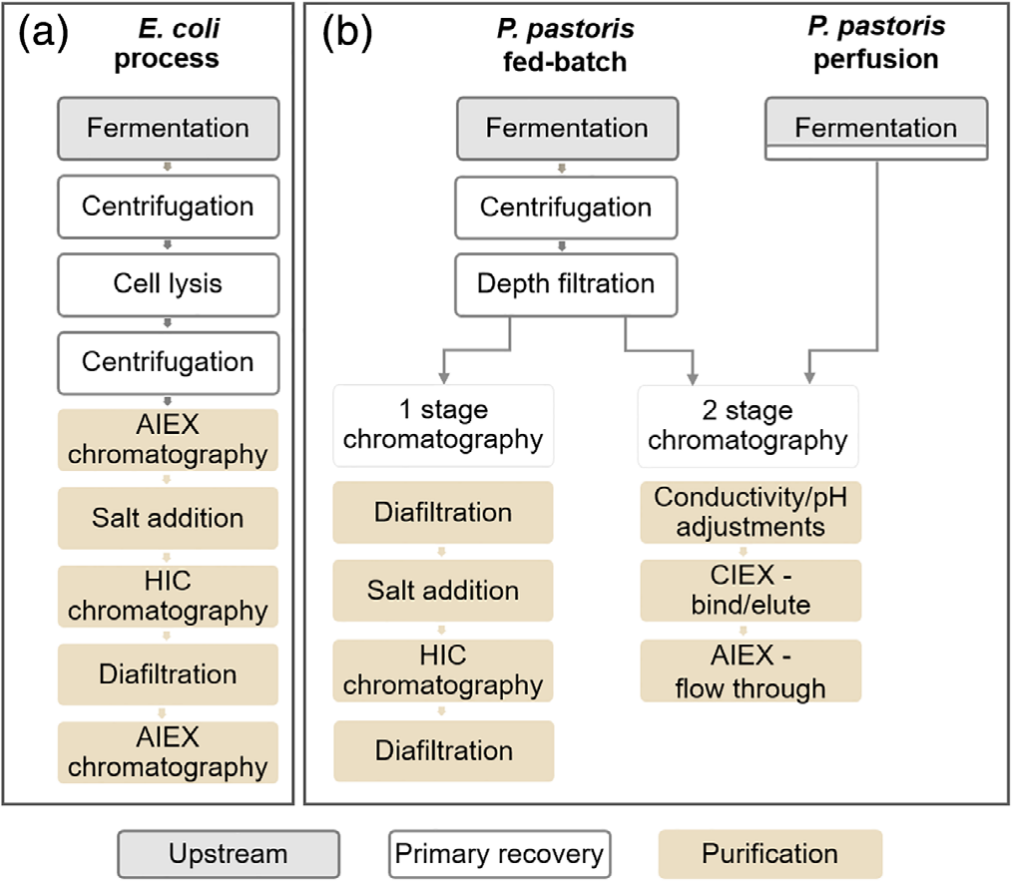
Schematic process for the production of NRRV expressed in *E. coli* and *P. pastoris*. NRRV production process (a) from product expressed in *E. coli* 9 and (b) *P. pastoris* process in fed‐batch or perfusion mode with 1 or 2 stage chromatography

Currently, these NRRV antigens are expressed intracellularly in *Escherichia coli*, with subsequent purification, requiring three chromatography steps (Figure 1a).^9^ Our aim is to move to a secreted system to significantly simplify this process, ultimately to be capable of incorporation into a single integrated process (Figure 1b),^1^ thereby reducing cost towards meeting the target of 15¢ per dose or less.

To simplify the production and purification process of NRRV, *Pichia pastoris*, a methylotrophic yeast, was used as expression system as it provides several advantages over *E. coli*. For example, *P. pastoris* can achieve high cell densities in fermentation, and it is capable of secreting high titers of correctly folded heterologous protein.^11^, ^12^ In addition, the secretion of endogenous proteins is very low, which is advantageous for the simplification of downstream processing (DSP).^13^, ^14^

One potential drawback of using *P. pastoris* as an expression system for heterologous proteins is the presence of proteolytic enzymes.^15^, ^16^ These can have detrimental effects, such as product degradation or truncation, leading to reduced yield, and loss of biological activity.^15^, ^17^, ^18^ Several approaches have been used to minimize the damage caused by proteolytic activity. At a fermentation level, supplementation with casamino acids and yeast peptone (substrate competition),^18^ addition of protease inhibitors,^19^, ^20^ changes in fermentation parameters such as reduction of pH and temperature,^12^, ^15^, ^21^, ^22^ as well as utilization of alternate carbon sources, and optimization of induction times have all been used to reduce proteolytic degradation.^17^ However, a generalized approach to solving problems of proteolytic degradation has not been established and solutions are case‐by‐case. Any actions taken at an upstream level to mitigate proteolysis, such as addition of inhibitors or competitors, can have consequences for the subsequent downstream, which will have to be capable of removing any additional components or inhibitors.^23^ The introduction of changes to an already defined antigen designed to decrease susceptibility to proteolysis can have regulatory consequences.^24^ Therefore, the development of upstream processing (USP) conditions that result in minimal protease activity are of great importance in production of full‐length products.

**Figure 2:**
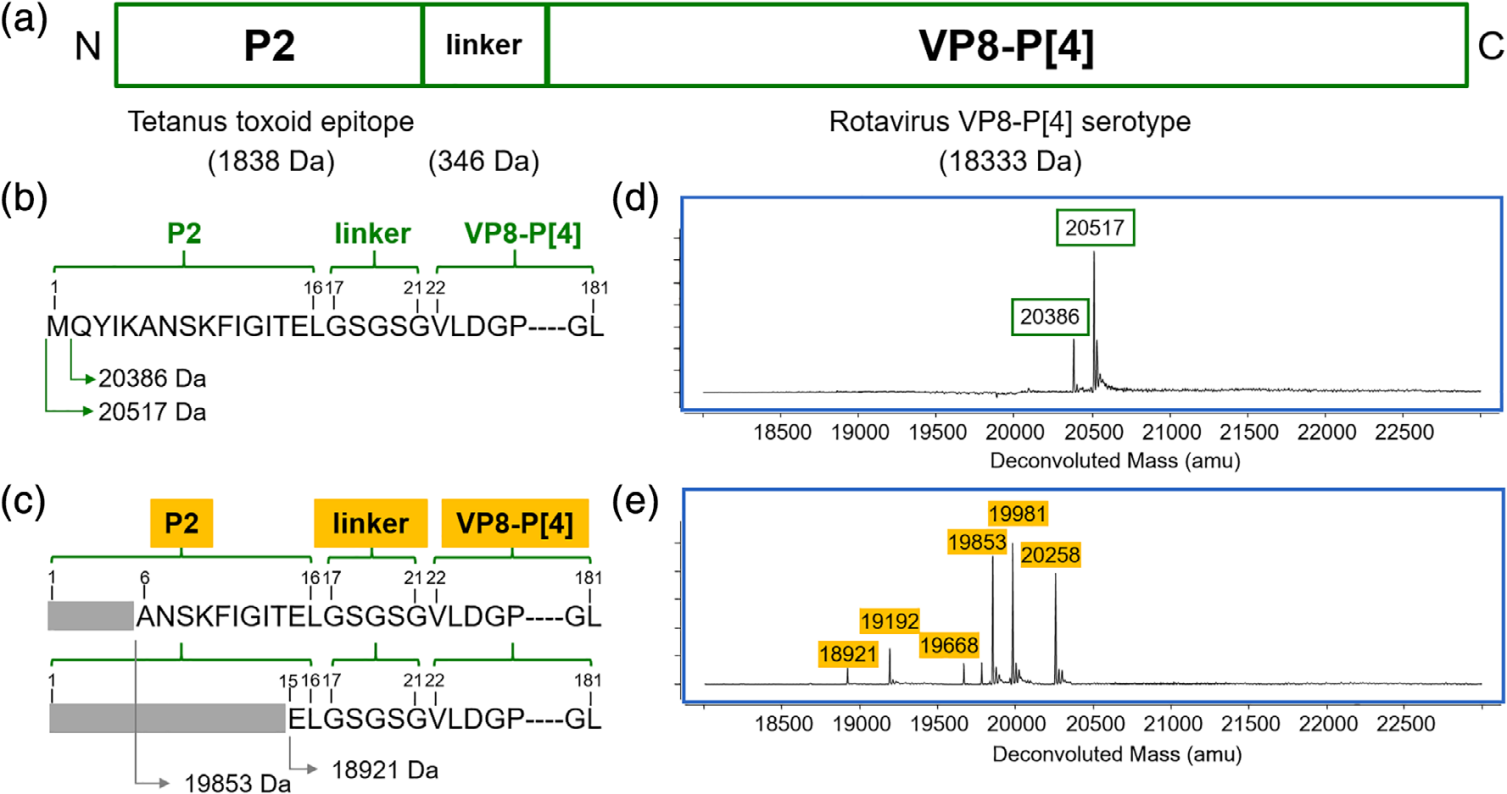
Schematic representation of NRRV (non‐replicating rotavirus VP8‐derived subunit vaccine) and its sequence with the corresponding intact mass spectrometry data. (a) Schematic representation of P2‐VP8‐P[4]. (b) Sequence for full‐length P2‐VP8‐P[4] and (c) different N‐terminal truncated species. Intact mass spectrometry data from fermentation samples of *P. pastoris* P[4], data edited to illustrate: (d) full‐length P2‐VP8‐P[4] and (e) different N‐terminal truncated species

In this study, a whole process approach was taken to identify compatible upstream and downstream conditions, where product quality was improved in fermentation and maintained throughout the purification process, to produce the highest proportion of full‐length NRRV (Figure 2).

## 2 MATERIALS AND METHODS

Chemicals, unless specified otherwise, were obtained from Sigma Chemical Co. Ltd. (Poole, Dorset, UK).

### 2.1 Cells and seed cultures

Fermentations were performed with *Pichia pastoris* (*Komagataella phaffii* NRRL Y‐11430) secreting a P[4] or a P[8] serotype of a non‐replicating rotavirus VP8‐derived subunit vaccine under control of the AOX1 promoter (a gift from the Love Lab at the Koch Institute at MIT) were re‐streaked from a frozen clonal stock onto solid yeast extract peptone dextrose media. A single colony was picked and expanded in buffered glycerol complex medium (BMGY) to create working cell banks (WCW).

Inocula from WCW were grown in BMGY medium incubated at 30°C for fermentations in Basal Salt Medium (BSM)^25^ or 25°C for fermentations in Rich Defined Medium (RDM),^26^ 250 rpm until they reached an OD_600_ ~10.

### 2.2 Fed‐batch fermentation

Fermentations were carried out using an ambr® 250 modular microbial system (Sartorius Stedim Biotech, Royston, UK). The bioreactors and integral reservoirs were aseptically filled with sterile media and feed solutions. Fermentations were carried out following Invitrogen's protocol for Mut^+^ cells.^25^ The initial fermentation volume was 120 mL of BSM with 0.52 mL of PTM_1_ trace salts. The operating conditions were 30°C, 30% DO (using air or a mix of air and oxygen when required at 0.5 vvm), pH 5.00 ± 0.15 (controlled with 10% [v/v] ammonium hydroxide), and antifoam (polypropylene glycol 2000) was automatically added by the system when required. Reactors were inoculated via the septum to an OD_600_ of 1.

Measurements from off‐gas CO_2_ sensors in the ambr250 modular were used by the system to calculate the carbon evolution rate (CER). A 20% drop in CER was used to detect the end of batch phase and automatically started the glycerol feed (18.15 mL/hr per liter of the initial fermentation volume [L_i Vol_]). Followed by a methanol adaptation phase using feed rate of 3.6 mL/hr/L_i Vol_. A decrease in CER during methanol adaptation indicated depletion of glycerol and was used as signal by the system to increase methanol feed rate, using an incremental step profile programmed into the ambr250 software (7.3 mL/hr/L_i Vol_ for 2 hr and 10.9 mL/hr/L_i Vol_ for the remainder of the fermentation). Glycerol and methanol feed rates were those specified by Invitrogen's protocol for Mut^+^ cells grown at 30°C.^25^

At the end of fermentation, the cell broth was centrifuged at 10,000 × *g* for 15 min and the supernatant was filtered using 0.80 μm cellulose nitrate and 0.45 μm polyvinylidene difluoride membrane filters (GE Healthcare Life Sciences, Buckinghamshire, UK). Samples were aliquoted into 20 or 30 mL and stored at −20°C until further processing.

### 2.3 Fermentation optimization

**Figure 3:**
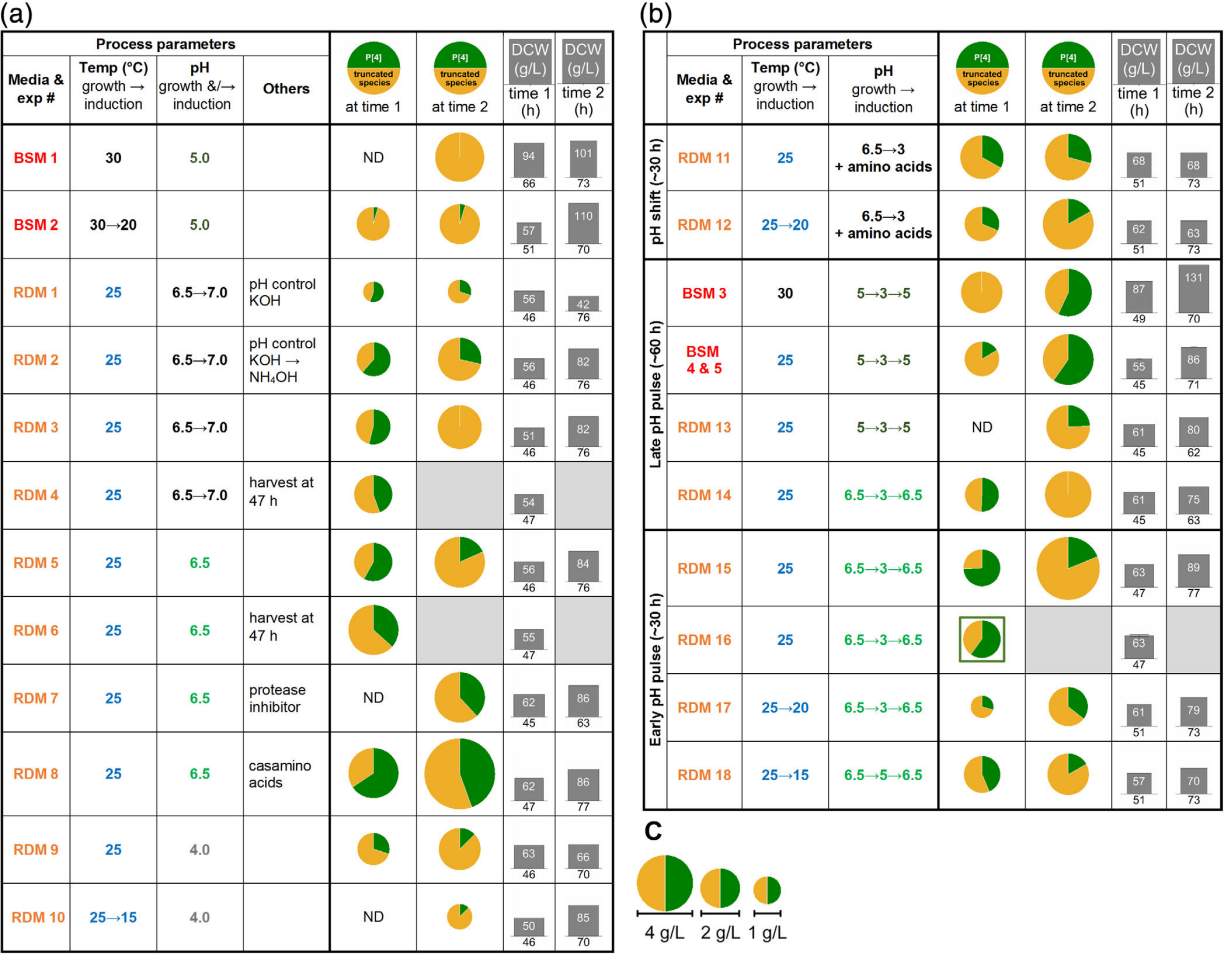
Fed‐batch fermentation parameters tested for *P. pastoris* expressing a NRRV. Proportion of full‐length P2‐VP8‐P[4] and truncated species determined by intact mass spectrometry and dry cell weight (DCW) at two different time points post‐inoculation. (a) Fermentations carried out at the indicated pH, (b) fermentations carried out with a pH pulse or pH shift, (c) scale for P2‐VP8‐P[4] concentration, the area of the circle showing full‐length and truncated P2‐VP8‐P[4] species (a and b) is proportional to its concentration in the supernatant

Fermentation parameters for batch and glycerol fed‐batch phases for BSM (30°C, 30% DO, pH 5.00 ± 0.15) and RDM (25°C, 25% DO, pH 6.50 ± 0.15) remain constant throughout the different fermentations, except for the fermentations listed in Figure 3a where the pH was changed (RDM 9 & 10). The pH was controlled with 10% (v/v) ammonium hydroxide, except for fermentations RDM 1 (Figure 3a) where it was controlled with potassium hydroxide solution (5 M) and RDM 2 with KOH during batch phase and NH_4_OH during fed‐batch phase.

The pH shift (Figure 3b, RDM 11 & 12) and early pH pulse (Figure 3b, RDM 15–18) were implemented at the end of methanol adaptation (~30 hr), and late pH pulse was implemented ~60 hr (Figure 3b, BSM 3–5, RDM 13 & 14). The reduction of the pH set point was achieved by turning off addition of base until the new pH set point was reached (~2 hr), held at pH 3.00 ± 0.15 for ~4 hr and then ramped for an hour to the higher set point using a programed function of the ambr250 software.

Induction temperature was reduced at the end of glycerol fed‐batch (Figure 3a and b, BSM 2, RDM 10, 12, 17, 18).

RDM 11 and 12 were supplemented throughout methanol adaptation and induction with amino acids (l‐glutamine, l‐arginine, and l‐asparagine) to a final concentration of 1 mM.

Supplementation of protease inhibitor (aprotinin) (Figure 3a RDM 7) or casamino acids (Figure 3a RDM 8) was done at the end of glycerol fed‐batch (~25 hr) to a final concentration of 3.9 μg/mL and 1% (v/v), respectively.^18^

For fermentations in BSM and RDM at 25°C methanol feed rate was 7.60 mL/hr/L_i Vol_ which corresponds to 70% of the methanol feed rate recommended in Invitrogen's protocol for Mut^+^ cells grown at 30°C,^25^ based on previous experience. Residual methanol concentration in the supernatant was measured off‐line (M&M Section 2.10). Methanol adaptation was done in incremental steps programed into the ambr250 software as follows: 1.16 mL/hr/L_i Vol_ for 4–5 hr until CER decreased (M&M Section 2.2) subsequently 2 hr steps of 2.0, 4.8, and 5.60 mL/hr/L_i Vol_. When induction temperature was reduced methanol feed rate was changed to 7.20 mL/hr/L_i Vol_ for BSM at 20°C (BSM 2), 5.30 mL/hr/L_i Vol_ for RDM at 20°C (RDM 12 & 17), and 3.75 mL/hr/L_i Vol_ for RDM at 15°C (RDM 10 & 18). Methanol adaptation feed rates were reduced by 30% for 20°C and 50% for 15°C.

### 2.4 Chromatography: resin selection

To assess the suitability of resins for use in bind, elute chromatography as the first stage in the purification process automated micro‐scale chromatography was carried out on a TECAN Evo liquid handling platform (Tecan UK Ltd, Reading, UK), using 96 well filter plates with either 2 or 20 μL per well (GE Healthcare Life Sciences)^27^ or 0.6 mL RoboColumns (custom packed by Repligen Europe B.V, Breda, The Netherlands).^28^ A total of 18 resins were screened for binding capacity and product quality (Table 1), determined by SDS‐PAGE. Resins from the following functional classes were screened: hydrophobic interaction (HIC), cation exchange (CIEX), and multimodal cation exchange (MM). HIC resins were screened for binding and elution at pH 4.0 and pH 7.5, over an ammonium sulfate concentration range of 0–1 M. CIEX and MM resins were screened over a pH range of 3.0–7.5 and a NaCl concentration of 0–1 M. Clarified fermentation medium was buffer exchanged for the appropriated binding buffer by diafiltration at 4°C using a 3 kDa cutoff regenerated cellulose membrane (Amicon Ultra 15, Merck Millipore, Watford, UK). Clarified medium was repeatedly diluted threefold and re‐concentrated to original volume. This was repeated six times until the buffer exchange was >99.5%. The best performing resin from each class was then scaled‐up.

**Table 1:** Affinity resins screened for potential first bind‐elute chromatography step

Resin class—HIC^a^		Resin class—CIEX		Resin class—MM	
Resin	Relative static binding capacity	Resin	Relative static binding capacity	Resin	Relative static binding capacity
**Capto phenyl (high sub)** b	**1.00**	**Capto S ImpAct**	**1.00**	**Nuvia cPrime**	**1.00**
Phenyl Sepharose 6 FF (low sub)	0.64	*Capto S* c	0.90	Capto MMC	0.32
*Capto butyl*	0.93	SP Sepharose FF	0.40	Capto MMC ImpRes	0.32
*Phenyl Sepharose 6 FF (high sub)*	1.10	*Capto SP ImpRes*	1.00	PPA Hypercel	0.30
Butyl‐S Sepharose 6 FF	0.46			Toyopearl MX‐Trp‐650 M	0.30
*Octyl Sepharose 4 FF*	0.89			*CMM Hypercel*	0.85
Butyl Sepharose 4 FF	0.66				
Capto Octyl	0.56				

a Resins from the following functional classes were screened; hydrophobic interaction (HIC), cation exchange (CIEX), and multimodal cation exchange (MM).

b The best performing resin from each class is indicated in **bold**.

c Resins shown *italicized* had adequate binding capacity but significant product truncation occurred during purification as determined by SDS‐PAGE and MS.

### 2.5 Hydrophobic interaction chromatography

HIC chromatography was performed on a 1 mL HiTrap Capto Phenyl (High Sub) column (GE Healthcare Life Sciences) at 1 mL/min attached to an AKTA Avant system (GE Healthcare Life Sciences). Purification was carried out in a base buffer of 50 mM Tris, pH 7.5 or 50 mM sodium citrate, pH 4.0. Clarified fermentation medium was buffer exchanged at 4°C using a 3 kDa cutoff regenerated cellulose membrane as in Section 2.4 into the appropriate pH buffer. This was brought to a final (NH_4_)_2_SO_4_ concentration of 1 M by addition of a 3 M (NH_4_)_2_SO_4_ in buffer stock solution. Bound protein was eluted by step‐wise decreases in (NH_4_)_2_SO_4_ concentration.

### 2.6 Multimodal chromatography

MMC was performed on 1 mL Nuvia cPrime columns (Bio‐Rad Ltd, Hertfordshire, UK) at 0.5 mL/min. Binding was carried out at pH 4.0 in 50 mM sodium citrate buffer. P2‐VP8‐P[4] was eluted at pH 6.0 in 50 mM sodium citrate buffer.

### 2.7 Cation exchange chromatography

CIEX chromatography was performed on 1 and 5 mL HiTrap Capto S ImpAct columns (GE Healthcare Life Sciences) at 1 or 5 mL/min on the AKTA Avant. Binding was carried out at pH 4.0 in 50 mM sodium citrate buffer. In initial experiments a gradient of 0–1 M NaCl in binding buffer was used as elution. However, to facilitate subsequent processing by AIEX without the need for a buffer exchange, a pH step to pH 7.5 in 50 mM Tris buffer was used to specifically elute P2‐VP8‐P[4].

### 2.8 Combined cation and anion exchange chromatography

A two‐step chromatography procedure was used for routine purification of all NRRV products. This was developed to maximize product purity and quality while minimizing the requirements for buffer exchange and thus ease of incorporation into a future integrated production system.

Clarified fermentation medium was brought to pH 4.0 by addition of 0.1 M citric acid. Conductivity was then adjusted to 10 mS/cm by addition of 50 mM sodium citrate, pH 4.0 buffer. This allowed for direct binding onto the Capto S ImpAct column. HCP were removed by a wash of 0.2 M NaCl in 50 mM sodium citrate, pH 4.0. Following removal of NaCl, a step of 50 mM Tris, pH 7.5 was employed to elute P2‐VP8‐P[4]. This eluate was then applied directly to a 5 mL HiTrap Capto Q ImpRes column (GE Healthcare Life Sciences) pre‐equilibrated in 50 mM Tris, pH 7.5. P2‐VP8‐P[4] was recovered in the flow‐through, with remaining HCP and DNA binding to the column.

### 2.9 Intact mass analysis of fermentation and purified NRRV samples

Before intact mass analysis, all NRRV fermentation samples were freshly thawed and buffer exchanged three‐times at 4°C to PBS buffer pH 7.2 using Amicon™ Ultra‐4, 10 kDa MWCO centrifugal filters (EMD Millipore) to remove fermentation media. Both purified and buffer‐exchanged fermentation samples were centrifuged at 14,000 × *g* for 1 min, and the top ~80% of the centrifuged protein sample was transferred to an HPLC vial. Intact mass analysis was performed using a 1220 LC system (Agilent Technologies, CA) connected in‐line to a 6230B time‐of‐flight mass spectrometer (Agilent Technologies) with typical injection volumes consisting of 0.5–5 μL (~15–20 pmol). Fermentation and purified protein samples were desalted using BEH C4 guard column, 2.1 × 5 mm, 1.7 μm (Waters Corporation, MA) and ZORBAX 300SB C3 column (Agilent Technologies), respectively. The LC gradient consisted of 20–90% B (A: water + 0.1% formic acid, B: acetonitrile + 0.1% formic acid) over 2 min at a flow rate of 0.8 mL/min for fermentation samples, and 20–70% B over 1 min at a flow rate of 1.5 mL/min for purified samples. Elution of NRRV proteins was monitored using the absorbance signal at 214 nm. One hundred microliters of isopropanol was injected after each sample to minimize carryover effect. The typical electrospray ionization parameters consisted of: 290°C gas temperature, 4,000 V *V*_cap_, and 275 V fragmentor. Mass spectra were collected from 700 to 2,800 m/z at 1 spectra/sec. Mass spectra were processed using MassHunter (Agilent Technologies), and the deconvolution range consisted of 10–50 kDa, with 1 Da mass step.

### 2.10 Analytical methods

Cell growth was measured off‐line using optical density measurements at 600 nm, wet, and dry cell weight (WCW, DCW).

Concentrations of glycerol and methanol in fermentation supernatant were determined off‐line using a Dionex UltiMate 3000 ultrahigh performance liquid chromatography with refractive index detector (Thermo Scientific, Loughborough, UK) with Aminex HPX‐87H column (Bio‐Rad, Hertfordshire, UK).

Protein concentration from culture supernatant and purification fractions was quantified using BCA assay (23225 Pierce™ Thermo Fisher Scientific, Loughborough, UK) and SDS‐PAGE 12% Bis‐Tris protein mini gels as per manufacturer's instructions (NuPAGE™, Thermo Fisher Scientific) using MOPS as running buffer. The proportion of heterologous proteins was determined by densitometry using Image Quant software (GE Healthcare Life Sciences).

Proteases in fermentation and purified samples were detected using Novex™ 10% Zymogram plus protein gels with gelatin (Thermo Fisher Scientific) as per manufacturer's instructions (incubating at 37°C, 24 hr).

Host cell protein (HCP) concentration of purified samples was determined using *P. pastoris* HCP ELISA kit (F140 Cygnus, Southport, NC) as per manufacturer's instructions. Values were calculated from three sample dilutions and expressed as parts‐per‐million (ppm). The cut‐off level for acceptable purity was set at a HCP level of 500 ppm.

DNA concentration of purified samples was determined using a Quant‐iT dsDNA high sensitivity assay kit (Thermo Fisher Scientific) as per manufacturer's instructions. For comparative purposes, DNA contamination levels in purified product is expressed as ng or pg DNA per dose, using the highest dose amount of 60 μg, currently under clinical evaluation.^9^, ^10^

## 3 RESULTS AND DISCUSSION

### 3.1 Identification of product truncation during fermentation

A NRRV subunit vaccine was expressed in *P. pastoris* using an α‐mating factor secretion signal, resulting in the protein of interest being secreted from the cells. Molecules of 20,517 and 20,386 Da were both considered as full‐length P2‐VP8‐P[4], as the methionine (131 Da) on the N‐terminal was an artifact of cloning from *E. coli* to *P*. *pastoris* (Figure 2b and d). Truncated species of P2‐VP8‐P[4] were defined when any other amino acid residues of the N‐terminal end were missing (Figure 2c and e).

Following Invitrogen's protocol, cells were grown in fed‐batch mode and induced with methanol.^25^ WCW achieved in fermentation BSM 1 was within the range expected (101 g/L);^25^ however, intact mass spectrometry analysis showed that there were mainly N‐terminal truncated species (Figure 3a). Reduction of induction temperature is known to improve yields of recombinant proteins; in this case, the induction temperature was reduced from 30 to 20°C (BSM 2), this did not impact the protein yield, and only a small proportion of full‐length P2‐VP8‐P[4] was observed at the two time points analyzed.

The tetanus toxoid P2 epitope has been shown to improve the immunogenicity of VP8 proteins (Figure 2a), and truncations of the P2 region could potentially lead to lower efficacy.^29^ To reduce the proportion of N‐terminal P[4] truncated species, we investigated the fermentation parameters (medium, pH, temperature, and harvest point) and purification sequence to produce full‐length P2‐VP8‐P[4] (Figure 2a and b).

### 3.2 Impact of media and pH control

Initial fermentations were carried out using a rich defined media (RDM), and pH was controlled with potassium hydroxide (Figure 3a, [RDM 1]).^26^ Cell growth and protein production were lower than in BSM 1 and 2, which could have been caused by nitrogen limitation.^30^ Despite the low titer (0.55 g/L), product quality was much improved to 55% full‐length P2‐VP8‐P[4] at 46 hr; however, it was not stable over time, decreasing to 20% at 76 hr. Ammonium hydroxide was used to control pH either during induction (RDM 2) or throughout the whole fermentation (RDM 3 & 5).^31^ Both strategies proved to be an efficient way to overcome the limitations in cell growth and protein production observed in RDM 1. To ease bioreactor operation, NH_4_OH was used to control pH throughout the whole fermentation in all further experiments. In changing pH from 6.5 to 7.0 (RDM 3) again, approximately 55% full‐length P2‐VP8‐P[4] was observed at 46 hr; however, this was not stable, and by 76 hr, P2‐VP8‐P[4] was fully truncated.

### 3.3 Identification of product truncation

It is known that proteases can be problematic during the production of heterologous proteins in *P. pastoris*.^17^, ^18^ To verify if proteases were the cause of product truncation over culture time, two approaches were taken: addition of protease inhibitor (aprotinin [RDM 7])^19^, ^20^ or substrate competition (casamino acids [RDM 8]).^18^ Both strategies were successful, and at harvest (77 and 63 hr, respectively), the proportion of full‐length P2‐VP8‐P[4] was ~40%. However, the use of protease inhibitors can be detrimental for cell growth, costly when scaling up, and have regulatory challenges.^23^ Supplementation of casamino acids can be challenging for USP and DSP, due to the variability in composition and the introduction of complex components (animal origin) to defined media.^30^ For these reasons, we chose not to modify the media but focus on USP control strategies to inhibit protease activity.

### 3.4 pH and temperature to control product truncation

Reduction of pH and temperature during fermentation have been used to decrease protease activity in fermentation supernatant.^12^, ^15^, ^21^, ^22^ Therefore, we tested these strategies to reduce protease activity and therefore product truncation to improve product quality.

Low pH strategies were tested: pH 4.0 throughout the fermentation (RDM 9 & 10) or a pH reduction strategy during induction from pH 6.5 to pH 3.0 (RDM 11& 12) with amino acid supplementation to avoid nitrogen limitation.^26^ Fermentations carried at pH 4.0 (RDM 9 & 10) showed ~10% full‐length P2‐VP8‐P[4] at harvest (70 hr). Fermentations with pH shift (RDM 11) showed ~30% full‐length P2‐VP8‐P[4] at 51 hr, and this proportion remain constant until harvest (70 hr).

Both low pH strategies were at carried out at 25°C or with a temperature reduction during induction (25 → 15°C [RDM 10] and 25 → 20°C [RDM 12]). In both cases, reducing induction temperature did not increase the proportion of full‐length P2‐VP8‐P[4]; on the contrary, when temperature was reduced to 15°C, the proportion of full‐length decreased to 15% (RDM 18). In our case, reducing pH during methanol induction was observed to be a favorable strategy to lower proteolytic activity and thereby increasing the proportion of full‐length P2‐VP8‐P[4].^12^, ^21^ However, further improvement was necessary to increase this proportion. Therefore, a novel strategy was designed where pH was reduced over a period of time by utilizing cell metabolism acidification when stopping the addition of base. The pH was held low for 1–3 hr and then increased to the original set point (BSM 3–5 and RDM 13–18). Under certain circumstances, this proved to be a successful strategy; for fermentation RDM 15 at 46 hr, 75% full‐length was observed; however by 77 hr, the proportion of full‐length P2‐VP8‐P[4] decreased to 20% (Figure 3b).

A reduction of temperature in combination with a pH pulse was tested in an attempt to maintain the highest proportion of full‐length P2‐VP8‐P[4]. However, reducing induction temperature did not improve product quality (RDM 17 & 18) (Figure 3b). When induction temperature was reduced to 15°C, due to low cell metabolism fermentation, the pH only reached 5 instead of 3. The pH pulse strategy was also tested with BSM (BSM 3) where 60% of the product was full‐length P2‐VP8‐P[4] (Figure 3b), which was a significant improvement on fermentation BSM 1 where only truncated species were present (Figure 3a).

**Figure 4:**
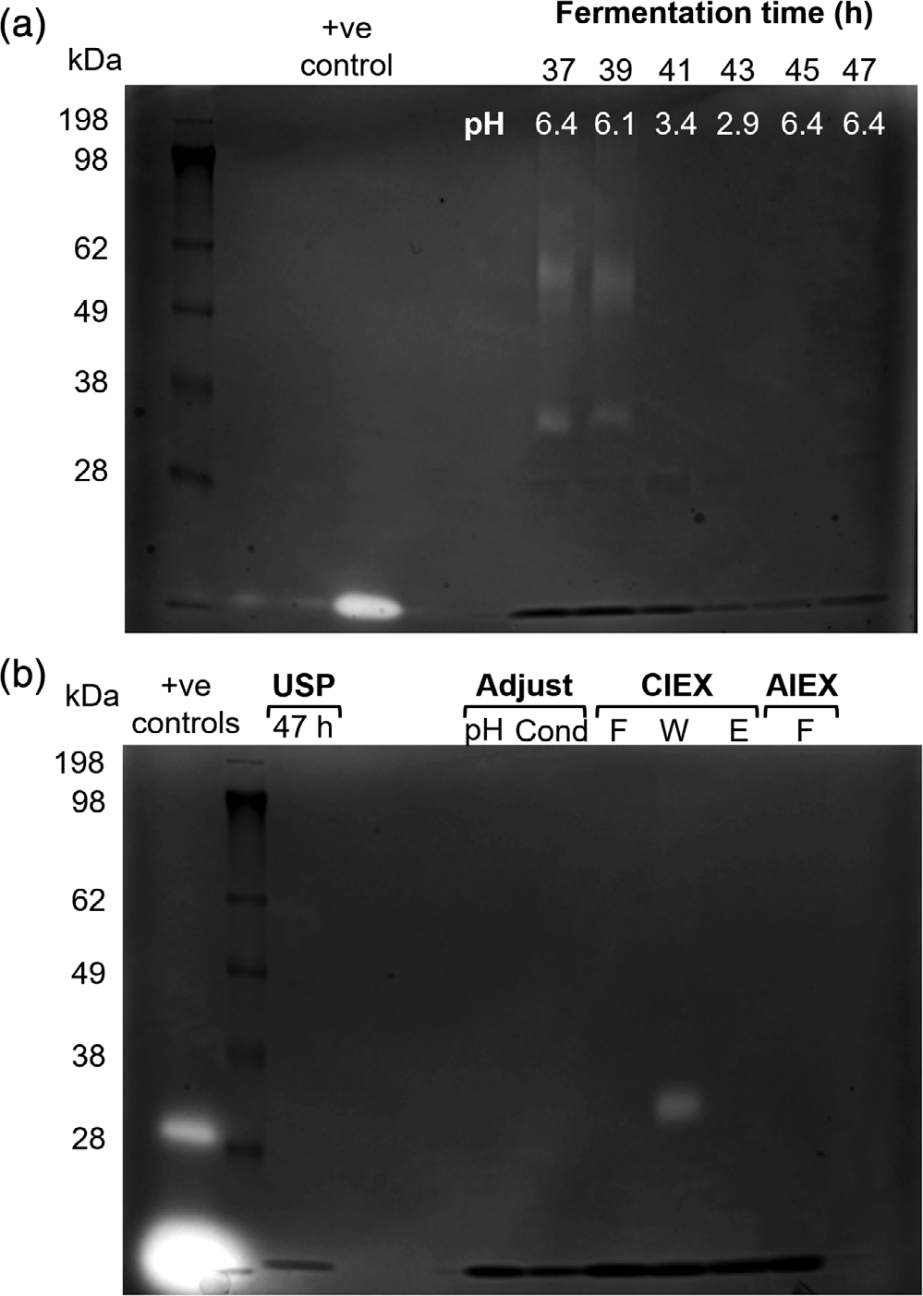
Zymograms from supernatant of *P. pastoris* fermentation and purified samples. (a) Samples from fermentation supernatant of P2‐VP8‐P[4] in RDM with pH pulse (RDM 16) from 6.5 → 3.0 → 6.5 between 38 and 45 hr. (b) Fermentation harvest after a freeze thaw cycle (USP 47 hr); after pH adjustment to pH 4 (pH) and conductivity (Cond); CIEX samples flow through (F), 0.2 M NaCl wash (W), and pH 7.5 elution/AIEX input (E) and AIEX flow through (F). Positive controls: thermolysin (37 kDa) and trypsin (19 kDa)

Zymograms were used to detect the presence of proteolytic enzymes in the fermentation supernatant throughout methanol induction (RDM 12) (Figure 4a). Protease activity at ~60 and ~40 kDa were visible at 37 and 39 hr when pH was greater than 6.1. When pH was reduced to 3.4, proteolytic activity was reduced and was neither detectable in the zymogram nor when the pH was increased to 6.4 (45 to 47 hr). Suggesting that a decrease on pH during methanol induction was an efficient strategy to reduce proteolytic activity in fermentation supernatant.

Based on these findings, a pH pulse during methanol induction was proposed and tested with BSM and RDM, leading to 60 and 75% full‐length P2‐VP8‐P[4], respectively (Figure 3b). However, depending on the media used, the reduction in pH was conducted at different times during the fermentation, at 60 and 30 hr for BSM and RDM, respectively (BSM 4 & 5 and RDM 15; Figure 3b).

### 3.5 Effect of fermentation time on product truncation

For most of the conditions tested, it was found that of the variables tested fermentation time had the greatest impact on product quality, as the proportion of truncated species increased overtime.^17^ For example, for RDM 15 (Figure 3b), P2‐VP8‐P[4] concentration at 46 hr was 1.7 g/L of which 75% is full‐length (1.25 g/L), and at 77 hr, protein production increased to 4.8 g/L but only 20% was full‐length (0.96 g/L), showing that longer induction was not beneficial. Therefore, an early harvest point was chosen where a greater proportion of full‐length protein was present in the supernatant, as the presence of closely related product impurities in harvest material would likely be challenging for DSP.

In the cases where the pH pulse was carried out, there was a short window to harvest before the product becomes truncated, this was approximately 3 hr after pH reached the upper set point; longer induction times lead to loss in full‐length P2‐VP8‐P[4] as seen in RDM 15. Alternative to avoid product truncation after holding pH at the high set point for 3 hr, temperature was reduce to 6°C, pH 4.0 and held low overnight. This was tested with P2‐VP8‐P[8], and the proportion of full‐length P2‐VP8‐P[8] was similar to when harvested 3 hr after the end of pH pulse, data not shown.

From all the conditions tested, three fermentation strategies led to the highest proportion of full‐length P2‐VP8‐P[4]. These were: (A) RDM at pH 6.5 or 7.0, harvest at 47 hr (RDM 4 & 6, respectively), (B) RDM with pH pulse (~30 hr) and harvest at 47 hr (RDM 16), and (C) BSM with pH pulse (~60 hr) and harvest at 70 hr (BSM 3) (Figure 3b). The purification of P2‐VP8‐P[4] produced by these three fed‐batch fermentation strategies presented different challenges to DSP.

### 3.6 Resin screening

We aimed to use a rational design approach to the development of DSP protocols, leading to the simplest process possible, ultimately being capable of incorporation into an integrated process (Figure 1b).^1^ To this end, combinations of chromatography elements were designed in such a way as to eliminate any intermediate buffer exchanges.

To select the best performing matrix for P2‐VP8‐P[4] purification, a total of 18 resins were tested in a combination of static binding and dynamic binding studies using automated 96‐well filter plate assays^27^ and 0.6 mL RoboColumns^28^ (Table 1). Resins were tested to define optimal binding and elution conditions for both pH and ionic strength. Resin suitability was also determined by product quality using SDS‐PAGE to assess the level of truncation during the purification. The best performing resin from each of the classes tested were then scaled up to 1 and 5 mL columns for further optimization. These were: HIC – Capto Phenyl (High Sub); multimodal – Nuvia C Prime; cation exchange – Capto S ImpAct.

### 3.7 Hydrophobic interaction chromatography

**Figure 5:**
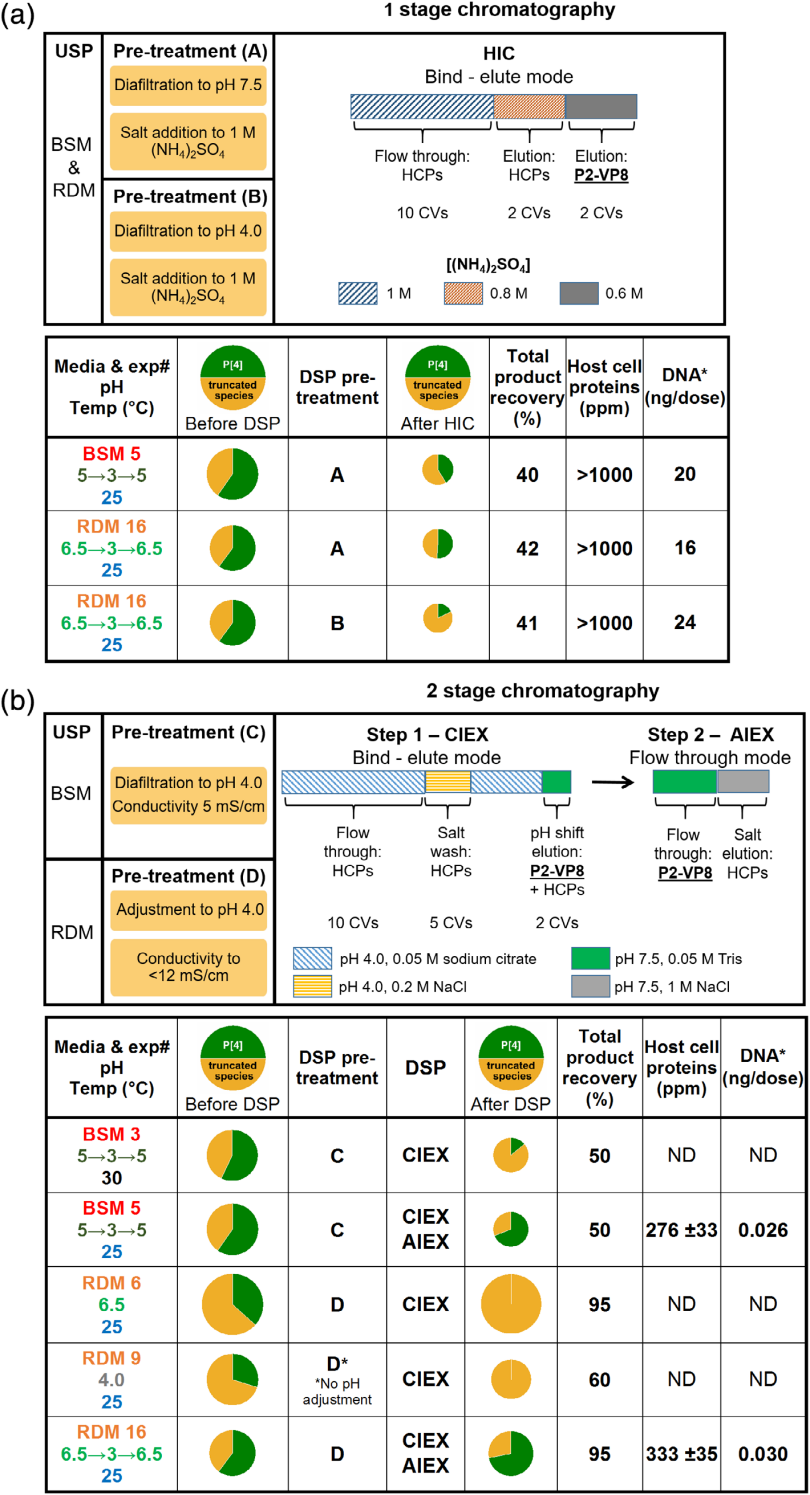
Downstream processing and purification of NRRV from best performing fed‐batch fermentations. Proportion of full‐length P2‐VP8‐P[4] and truncated species determined by intact mass spectrometry. Required pre‐column treatment is shown and bind‐elution conditions indicated. Product recovery was determined by mass balance calculation using densitometry of SDS‐PAGE. HCP and DNA content were determined for final purified NRRV (using the highest dose amount of 60 μg, currently under clinical evaluation). (a) One‐stage chromatography by HIC. (b) Two‐stage chromatography by CIEX followed by AIEX. For cases were insufficient levels of full‐length, NRRV was recovered after initial CIEX step no further processing was performed

**Figure 6:**
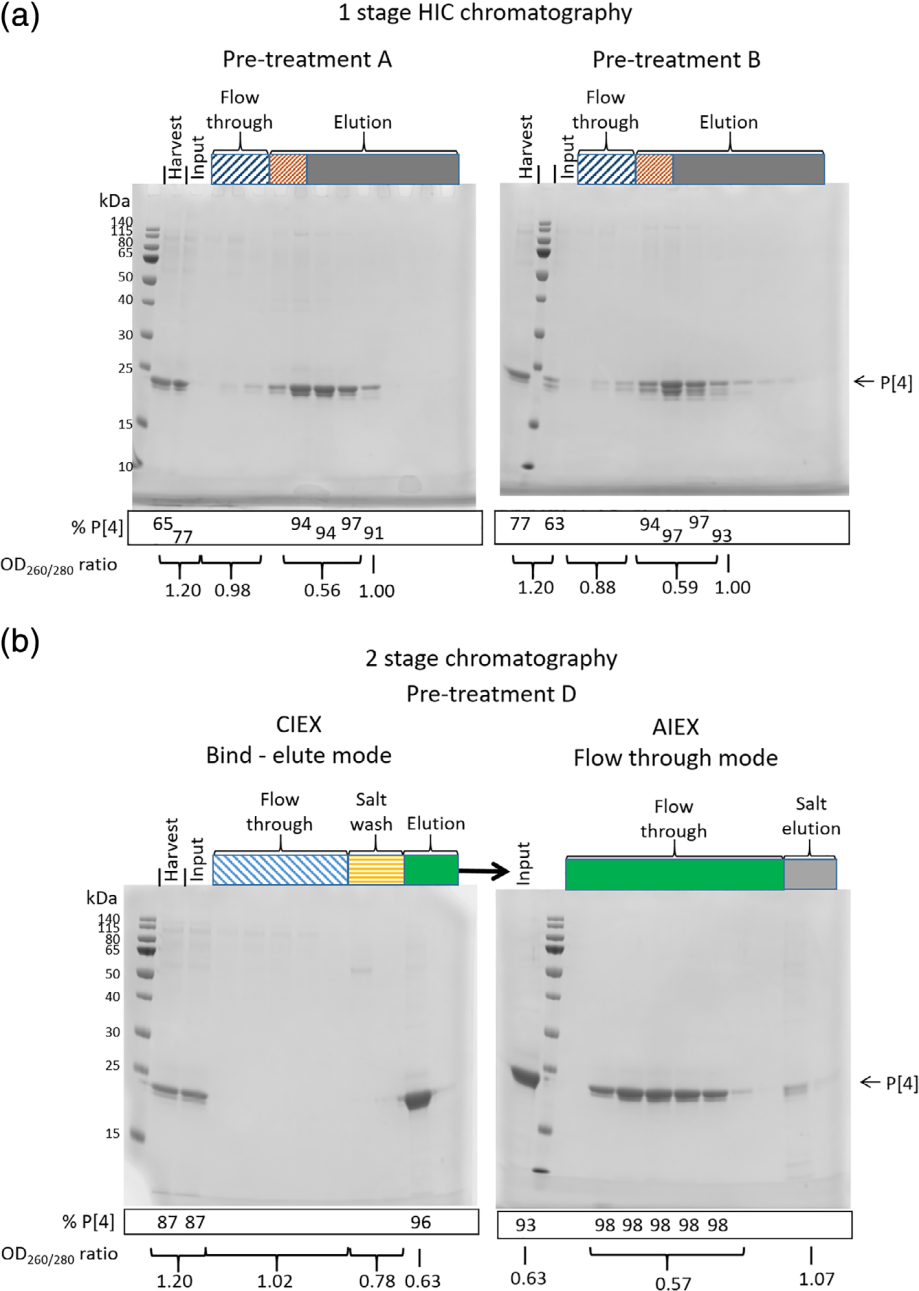
SDS‐PAGE analysis of downstream processing and purification of NRRV serotype P[4] from fed‐batch fermentation (RDM 16). Pre‐treatment and bind‐elute conditions are as defined in Figure 5. Input indicates material post pretreatment. The percentage of P2‐VP8‐P[4] was calculated by densitometry and the OD_260/280_ ratios measured directly from the column fractions. (a) One stage HIC chromatography at pH 7.5 (pre‐treatment A) and pH 4.0 (pre‐treatment B). (b) Two‐stage CIEX–AIEX chromatography from material using pre‐treatment D (pre‐treatment C gave identical results)

HIC is used as a bind‐elute step in the published protocol based on *E. coli* expressed material, after the removal of HCP by anion exchange.^9^ Therefore, it was expected that yeast expressed P2‐VP8‐P[4] could be purified by a bind‐elute strategy using HIC resins. This was indeed the case. However, to obtain efficient binding, the clarified medium must first be subject to buffer exchange and addition of 1 M ammonium sulfate. P2‐VP8‐P[4] is eluted by decreasing the ammonium sulfate concentration to 0.8 M (Figure 5a). Initially, clarified medium was buffer exchanged to Tris at pH 7.5 (pre‐treatment A, Figure 5a) as this was the binding condition shown to work for bacterial P2‐VP8‐P[4]. However, as shown in Figure 5a, bringing the pH to 7.5 leads to a high level of truncation of P2‐VP8‐P[4] by residual protease activity. This could decrease the yield of full‐length protein due to proteolysis during handling/purification. To alleviate this, clarified medium was instead buffer exchanged to a pH of 4.0 (pre‐treatment B, Figure 5a). This was found not to change the bind‐elute characteristics of P2‐VP8‐P[4] (Figure 6a). However, performing the chromatography at this reduced pH did not have the desired effect of reducing protease activity during purification, rather it resulted in a higher levels of truncation compared to P2‐VP8‐P[4] after HIC purification at pH 7.5 (Figures 5a and 6a). Use of HIC alone also resulted in unacceptably high levels of both HCP (>1,000 ppm) and residual DNA (16–24 ng/dose) irrespective of chromatography pH (Figure 5a). Although this protocol produced full‐length material, it was not stable to further proteolysis, becoming completely truncated within 4 days at room temperature. Showing that significant levels of protease remained after HIC purification (data not shown).

### 3.8 Cation exchange and multimodal chromatography

The HIC procedure while being significantly simplified over that used previously is still too complex for incorporation into a fully integrated process,^1^ due to the requirement for buffer exchanges. Ideally, a process that requires no buffer exchange to be performed between the steps is desirable. This situation is most easily met by the use of a cation exchange or multimodal resin as the capture step. Examples of both classes of resin were found to be able to purify P2‐VP8‐P[4] (Table 1). However, the use of a cation exchanger (Capto S ImpAct) was found to provide the most robust protocol. To achieve efficient binding, it is sufficient simply to adjust the clarified medium to pH 4.0 and conductivity to 10 mS/cm by direct addition of dilute citric acid (pre‐treatment D, Figure 5b). This removes the need for any buffer exchange between fermentation output and column purification, thereby increasing compatibility with an integrated USP‐DSP process.^1^ The residual protease, which also binds the column, is efficiently removed by a pre‐elution wash step at pH 4.0 with 0.2 M NaCl (Figure 4b). P2‐VP8‐P[4] is then eluted by increasing the salt to 0.4 M NaCl. The material produced by this single‐column purification was found still to contain residual protease activity, which was only detected by MS analysis of material stored at room temperature for 2 days. This revealed that further truncated species were being formed. To remove this activity and other residual HCP and nucleic acid impurities, a second anion exchange column (Capto Q ImpRes) was included in flow through mode (Figure 5b). This resulted in final HCP levels of <500 ppm and residual DNA of 26 pg/dose. To alleviate the need for a buffer exchange between cation and anion exchange columns, the elution procedure was modified such that following the pH 4.0, 0.2 M NaCl wash the column is brought back to 0 M NaCl. P2‐VP8‐P[4] is then eluted by elevating the pH to 7.5 (Figures 5b and 6b). This eluate is then applied directly to a Capto Q ImpRes column, where the HCPs and DNA are bound and the P2‐VP8‐P[4] is in the flow‐through (Figures 5b and 6b). This simplified protocol also resulted in final HCP levels <500 ppm and DNA levels of 30 pg/dose (Figure 5b) and is compatible with incorporation into an integrated USP–DSP methodology.^1^ Material produced in this manner was found to be stable at pH 7.5 for up to 8 days at room temperature when analyzed by intact MS (data not shown).

### 3.9 USP‐DSP interactions

**Figure 7:**
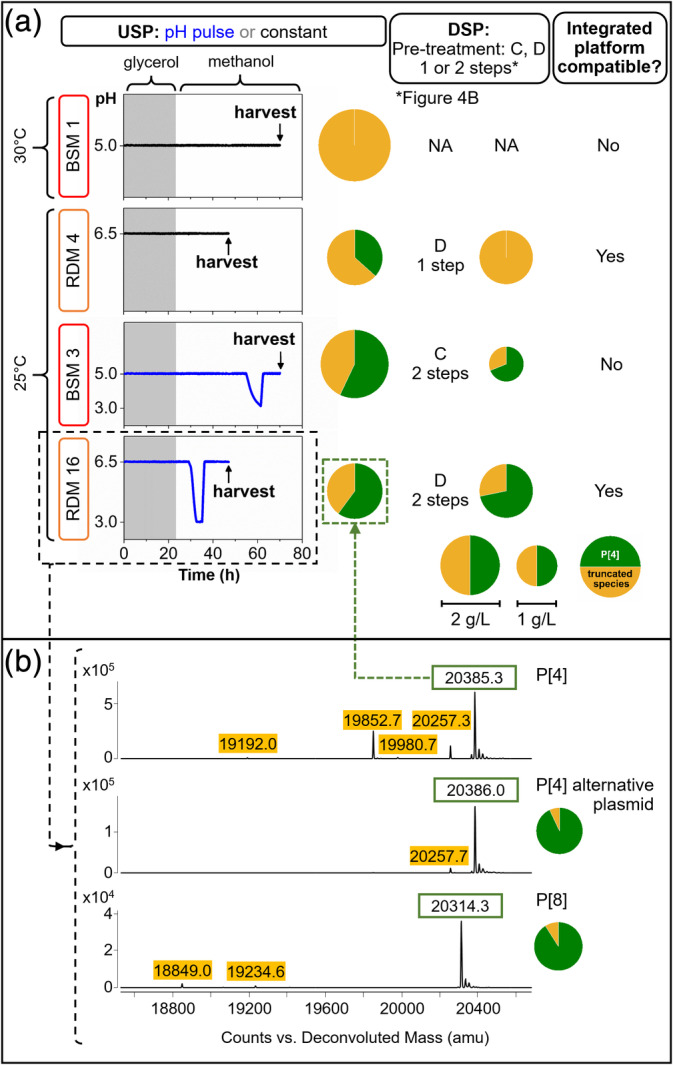
Comparison of optimized and standard fed‐batch fermentation (USP) and purifications processes (DSP) for the production of NRRV serotype P[4]. (a) pH traces for fermentations in BSM or RDM carried out at constant pH or with a pH pulse, with its corresponding proportion of full‐length and truncated P[4] at harvest and after CIEX and AIEX; DSP pre‐treatment (C or D) and number of chromatography steps see Figure 5b. (b) Intact mass spectrometry from fermentation samples of NRRV P2‐VP8‐P[4] and P2‐VP8‐P[8] serotypes

Having significantly improved the individual elements of both USP and DSP a whole process approach was used to identify the best integrated process to give the highest yield of full‐length P2‐VP8‐P[4] (Figure 7a). Irrespective of fermentation medium (BSM or RDM), the use of a pH pulse was required to produce high titers of P2‐VP8‐P[4] with the majority being full‐length (Figure 7a, [BSM 3, RDM 16]). However material generated using BSM must first undergo a buffer exchange to allow processing on a CIEX, this results in a 50% loss of P2‐VP8‐P[4] through the entire DSP (Figure 5b [BSM 3 & 5]). BSM fermentations at 25°C (BSM 5) and 30°C (BSM 3) both produce acceptable levels of full‐length P2‐VP8‐P[4]. However, when the material produced at 30°C is processed through a DSP, the resulting product is almost entirely truncated, with less than 10% full‐length, after buffer exchange and CIEX chromatography. This is not the case for material grown at 25°C which resulted in a final product being ~60% full‐length, after buffer exchange and both CIEX and AIEX chromatography. This most likely indicates higher levels of protease at the higher temperature (Figure 5b).

The use of RDM in fermentation removes the need for a buffer exchange before DSP. To produce a high titer of full‐length P2‐VP8‐P[4], fermentations require a pH pulse and a short induction period. The harvest only requires adjustment of pH and conductivity to allow it to be passed rapidly through the CIEX–AIEX chromatography. Maintaining 72% of full‐length P2‐VP8‐P[4] with a 95% recovery over the whole process.

### 3.10 Verification of the improved process

The improved fermentation protocol RDM 15 (RDM, 25°C, pH 6.5 → 3.0 → 6.5, harvest 3 hr after end of pH pulse [~47 hr]) (Figure 7a) was tested with P2‐VP8‐P[4] (RDM 16). At harvest, DCW was 63 ± 2.3 g/L, protein concentration of 2.17 ± 0.16 g/L (*n* = 4), and 60% of full‐length P2‐VP8‐P[4] (Figure 7b). The corresponding CIEX and AIEX (pre‐treatment D, Figure 5b) purified material had 72% full‐length product (Figure 7a).

There was a difference in the percentage of full‐length obtained from fermentations RDM 15 and RDM 16, 75% and 60%, respectively (Figure 3b), this might have been caused by batch to batch variability, and/or sample degradation during preparation for analysis. The difference was greater in the USP samples where there were proteases in the supernatant, the DSP samples from the same fermentation showed greater proportion of full‐length P2‐VP8‐P[4] (72%) suggesting that some proteolysis occurred during sample holding and preparation.

USP‐DSP protocols were tested with a third independent batch achieving 76% full‐length P2‐VP8‐P[4] determined by intact MS analysis with 81% total product recovery. In addition, an alternative P[4] expression plasmid which failed to yield any full‐length products under standard BSM fermentation conditions (Section 2.2) was also tested. Under the improved process, this produced 93% full‐length product (Figure 7b).

To serve as a platform process for the production of NRRV vaccine candidates, the USP‐DSP must be capable of producing full‐length product from multiple virus serotypes. This USP‐DSP protocol was successfully tested with cells expressing different product constructs P2‐VP8‐P[4] and P2‐VP8‐P[8]. The MS results showed that for both serotypes the greater proportion of product was full‐length being 72–93% for P[4] and 91% for P[8] (Figure 7b). Proving that it is feasible to use this process as a platform for the production of different NRRV antigens.

## 4 CONCLUSION

Proteases can be problematic during the production of secreted heterologous proteins in *P. pastoris*.^17^, ^18^ Here, we show that product truncation can be addressed at a process level to improve the quality of NRRV antigen. Fermentation process conditions have been improved to minimize proteolysis using a novel acidic pH pulse strategy. This has resulted in a fermentation harvest containing the majority of material as full‐length, as verified by intact mass spectrometry analysis. The use of RDM in fermentations removes the need for a buffer exchange before DSP. A two‐step chromatography process (CIEX and AIEX) removed proteases preventing further N‐terminal proteolytic degradation of the NRRV antigen. The resulting USP and DSP strategies are potentially compatible with incorporation into an integrated production platform for manufacture of clinical grade NRRV based vaccine on multiple serotypes.

For future studies, the identification of the specific proteases involved will help in the more rapid design and implementation of combined USP‐DSP protocols for other secreted products in *P. pastoris*. Although the two‐step chromatography used reduces HCP and nucleic acid levels to within specified tolerances, it is possible that removal of the small amounts of residual product related impurities would require inclusion of an additional step if this proves necessary for clinical grade vaccine.

## CONFLICT OF INTEREST

The authors declare no financial or commercial conflict of interest.
